# Machine Learning Analysis Reveals Distinct Neuroimaging Biomarker Patterns Across Ethnic Groups in Alzheimer's Disease

**DOI:** 10.1002/alz70856_096883

**Published:** 2025-12-24

**Authors:** Daniel C. Gibbs, Ben Black, Melissa Petersen, Leigh A. Johnson, James Hall, Sid E. O'Bryant, Fan Zhang

**Affiliations:** ^1^ Texas College of Osteopathic Medicine, University of North Texas Health Science Center, Fort Worth, TX, USA; ^2^ Institute for Translational Research, University of North Texas Health Science Center, Fort Worth, TX, USA

## Abstract

**Background:**

Alzheimer's Disease (AD) affects approximately 7 million Americans, with projections indicating this number will double by 2060. While neuroimaging has advanced AD research, understanding biomarker variations across ethnic groups remains crucial for developing effective diagnostic approaches.

**Method:**

Neuroimaging data was analyzed from 2,950 participants (22% African American [AA], 39% Hispanic, 39% Non‐Hispanic White [NHW]) in the Health and Aging Brain Study. The dataset included MRI cortical thickness measurements, diffusion tensor imaging (DTI) metrics, and PET scans. Using Support Vector Machine (SVM) with SHapley Additive exPlanations (SHAP), we evaluated feature importance across ethnic groups to distinguish between cognitively impaired (CI, *N* = 783) and cognitively unimpaired (CU, *N* = 2,168) individuals.

**Result:**

Model performance varied significantly across ethnic groups, with the highest accuracy in Hispanic participants (AUC=99.04%), followed by NHW (AUC=82.19%) and AA populations (AUC=75.44%). PET‐Tau Posterior Cingulate SUVR emerged as the most significant predictor across all groups. Secondary biomarkers showed ethnic‐specific patterns: R bankssts thickness in AA, L isthmuscingulate thickness in Hispanic, and AB FBB frontal SUVR in NHW populations.

**Conclusion:**

Our findings reveal distinct neuroimaging biomarker patterns across ethnic groups while highlighting the consistent importance of Tau‐PET measurements. The varying model performance suggests current neuroimaging biomarkers may have different sensitivities across populations. These results emphasize the need for ethnically sensitive diagnostic approaches and warrant further investigation into population‐specific AD manifestations. Future research should focus on validating these ethnic‐specific biomarker patterns through longitudinal studies while considering additional factors such as genetics and social determinants of health.

